# Bipolar Disorder and Multiple Sclerosis: A Case Series

**DOI:** 10.1155/2014/536503

**Published:** 2014-03-17

**Authors:** Youssef Sidhom, Mouna Ben Djebara, Yosr Hizem, Istabrak Abdelkefi, Imen Kacem, Amina Gargouri, Riadh Gouider

**Affiliations:** Department of Neurology, Razi Hospital, Manouba, 2010 Tunis, Tunisia

## Abstract

*Background.* The prevalence of psychiatric disturbance for patients with multiple sclerosis (MS) is higher than that observed in other chronic health conditions. We report three cases of MS and bipolar disorder and we discuss the possible etiological hypothesis and treatment options. *Observations.* All patients fulfilled the McDonald criteria for MS. Two patients were followed up in psychiatry for manic or depressive symptoms before developing MS. A third patient was diagnosed with MS and developed deferred psychotic symptoms. Some clinical and radiological features are highlighted in our patients: one manic episode induced by high dose corticosteroids and one case of a new orbitofrontal MRI lesion concomitant with the emergence of psychiatric symptoms. All patients needed antipsychotic treatment with almost good tolerance for high dose corticosteroids and interferon beta treatment. *Conclusions.* MRI lesions suggest the possible implication of local MS-related brain damage in development of pure “psychiatric fits” in MS. Genetic susceptibility is another hypothesis for this association. We have noticed that interferon beta treatments were well tolerated while high dose corticosteroids may induce manic fits.

## 1. Introduction

Emotional disturbances are highly prevalent with an early onset in patients with multiple sclerosis (MS) [1]. The presence of psychiatric symptoms in MS was underlined and systematically described as early as in 1877 by Charcot. However, it was only in the last two decades that more detailed studies were carried out [2]. Many of these symptoms are described and are not necessarily related to the psychological impact of such a chronic and disabling disease. Depression is the most common psychiatric manifestation with a prevalence of 22–54% [3]. Other manifestations are anxiety, euphoria, and psychosis [4]. Bipolar disorder and MS coexistence is not common but well proven. A few cases have been already reported [5–8]. The link between these two disorders is not fully determined. Herein, we present three cases of MS and bipolar disorder and we discuss the possible etiological hypothesis and treatment options.

## 2. Case Reports

### 2.1. Case  1

**Figure 1:**
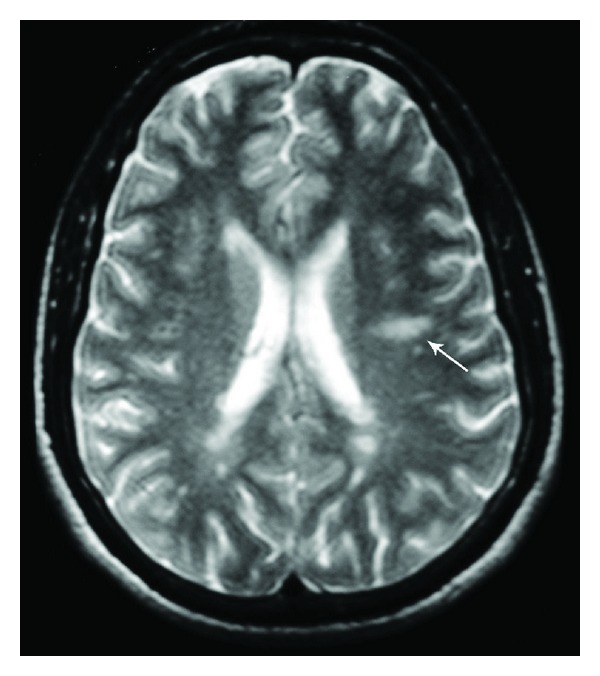
Axial cerebral T2-weighted image showing multiple lesions in periventricular and subcortical white matter. Dawson's finger (arrow) is a characteristic finding in multiple sclerosis.

A 39-year-old man, with no family medical history, was followed up since 1992 at the age of 20 for bipolar disorder with mainly manic fits. He was treated with a mood stabilizer (lithium carbonate). In October 2003, he presented a decrease in visual acuity that resolved spontaneously after 15 days. In September 2004, he reported paresthesia and weakness of the left side of the body associated with urinary incontinence. The symptoms regressed after a five-day course of intravenous methylprednisolone (1 g per day). Neurological examination revealed a left hemiparesis and left pyramidal syndrome. Cerebrospinal MRI showed multiple T2-weighted hyperintense lesions in periventricular white matter and in corpus callosum, as well as the cervical spine at C2 and C3 (Figure 1). Radiological Barkhof criteria for MS were fulfilled. Autoantibody (ANA, anti-DNA, anti-SSA, anti-SSB, and anti-SM) and serology (syphilis, hepatitis B and C, and HIV) tests were negative. Visual evoked potentials showed increased latencies. Based on these findings, the patient was diagnosed with relapsing-remitting MS, and interferon beta-1A treatment had been initiated since December 2004. During the seven years of follow-up, the patient presented two neurological fits in December 2005 and February 2007. His last EDSS score was 1. He repeatedly discontinued his mood stabilizer treatment and had concomitant manic fits.

### 2.2. Case  2

A 38-year-old woman, with a family history of bipolar disorder in a maternal uncle, has been followed up since the age of 20 for manic depressive psychosis with mainly manic episodes. A mood stabilizer treatment was prescribed (lithium carbonate). She consulted in 2005 about an episode of weakness of both lower limbs. She reported a similar episode in 2004 that resolved spontaneously after a few days. Neurological examination revealed a quadripyramidal syndrome with right kinetic cerebellar syndrome. Cerebrospinal MRI displayed multiple ovoid and confluent T2 hyperintense lesions in periventricular and semioval white matter and a cervical lesion at the level of C6. No gadolinium-enhanced lesions were identified. Serological tests for syphilis, hepatitis B and C, and HIV, inflammatory tests, and anti-nuclear antibodies were negative. The diagnosis of clinically definite MS was made. On follow-up, she presented one motor fit per year and was treated with high doses of methylprednisolone in each episode. The last fit was in June 2010. She consulted about an episode of weakness of both lower limbs. A three-day course of high doses of intravenous methylprednisolone was prescribed. Two days later, she presented a manic fit requiring hospitalization in a psychiatric department. Cerebrospinal MRI showed no new lesions. The patient was treated with atypical antipsychotics (olanzapine) associated with lithium carbonate with resolution of the manic episode.

### 2.3. Case  3

**Figure 2:**
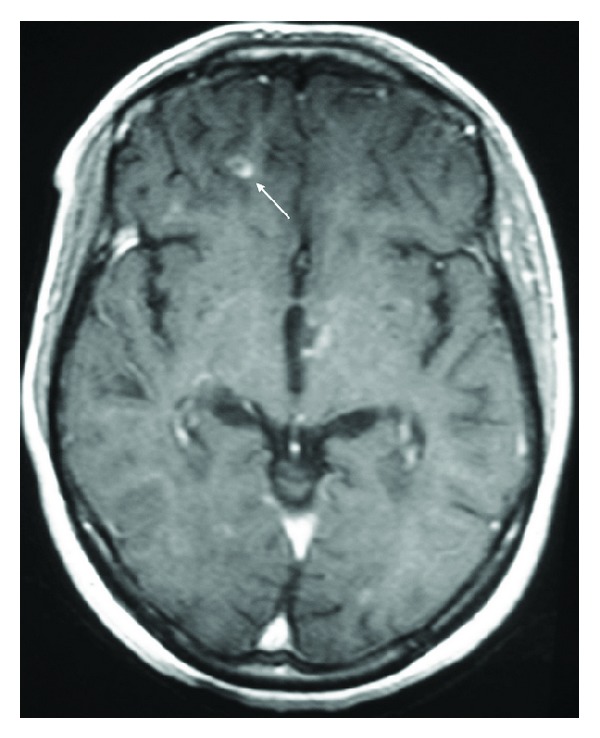
Axial cerebral T1-weighted image revealing a right orbitofrontal active lesion with gadolinium enhancement (arrow).

A 23-year-old woman, with a family history of bipolar disorder in a sister, had no past personal history. In April 2005, she presented clumsiness in the right side of her body that completely regressed after two months. In February 2007, she reported weakness in her left hemibody with blurred vision, completely regressed after one month. In November 2008, the patient was hospitalized for a motor deficiency in the right hemibody associated with diplopia and bladder dysfunction. Neurological examination showed right hemiparesis with quadripyramidal syndrome and static and kinetic cerebellar syndrome. Cerebral MRI revealed multiple T2 hyperintense lesions in periventricular and subcortical “white matter”, mainly in frontal and temporal lobes, right cerebellar peduncle, and corpus callosum and two cervical lesions at the level of C2 and C3. Analysis of the cerebrospinal fluid (CSF) detected the presence of oligoclonal bands. The diagnosis of relapsing-remitting MS was confirmed according to McDonald criteria. The patient received a 5-day course of intravenous methylprednisolone (1 g per day). Six months later, the patient developed psychiatric symptoms with irritability, frequent crying, social withdrawal, and insomnia. She has not consulted and received no treatment. A few months later, the clinical picture changed spontaneously and the patient was hospitalized for a manic episode with euphoria, grandiosity, hyperactivity, and reduced need to sleep. Neurological examination was normal. Cerebral MRI showed a right orbitofrontal active lesion with gadolinium enhancement (Figure 2). She was treated with an antipsychotic (haloperidol 15 mg t.i.d) in association with a mood stabilizer (sodium valproate 600 mg t.i.d) with resolution of the manic episode. An interferon beta-1A treatment was started in April 2011 with good tolerance.

## 3. Discussion

The first case report describes a man with a long term history of bipolar disorder who developed MS. In this case, the most likely hypothesis advanced to explain this comorbidity would be a casual association. In fact, MS is a relatively rare disease with an estimated prevalence in Tunisia of 20.1 per 100,000 inhabitants [9], yet bipolar disorder affects 1% of the population [10]. As patients with bipolar disorder have at least the same risk of developing MS as subjects not suffering from bipolar disorder, it is possible that both conditions coexist with no direct link between them. However, Joffe et al. have shown that manic depressive psychosis appears to be significantly more common in MS patients than in the general population, prompting the search for alternative hypotheses [11]. Etiopathogenic bases explaining this association are not yet understood. One hypothesis is that mood disorders could be an inaugural manifestation in MS [12] or may be the presenting symptom of MS years before the development of neurological signs as shown in the first two patients [13]. In fact, Lyoo et al. performed brain MRI on 2783 patients who were referred as part of their psychiatric evaluation. Their findings indicated that 0.83% of the patients had T2-weighted white matter hyperintensities consistent with MS, which was almost 15 times the reported prevalence of MS in the general population in the United States [14]. These results suggest the possibility of pure “psychiatric fits” in MS. This hypothesis has been reported by several authors [15, 16]. However, a study of 7301 autopsies of patients followed in psychiatry has led to the anatomopathological confirmation of MS in 14 patients, none of whom has a pure psychiatric form without any associated neurological manifestations [17].

The second case report illustrates the occurrence of a manic episode following high doses of methylprednisolone. Corticosteroids have long been implicated in the precipitation of the onset of certain psychiatric symptoms. The most common adverse effects of short-term corticosteroid therapy are mood disorder, euphoria, and hypomania. Conversely, long-term therapy tends to induce depressive symptoms [18]. Dosage is directly related to the incidence of adverse effects but is not related to the timing, severity, or duration of these effects [19]. Among MS patients treated with corticosteroids or ACTH, two systematic studies reported that 40% became depressed, 31% hypomanic, and 11% developed a mixed state and 16% a psychotic state [20]. Interestingly, these symptoms do not occur with every drug exposure and appear more frequently in case of a discontinuous treatment [21].

Although research of the etiopathogenesis of the association between MS and bipolar disorder is still limited, a common genetic susceptibility to both diseases was discussed. In a series of 56 patients, Schiffer et al. noted a higher frequency of the HLA-DR2 and -DR3 haplotype and a decrease in the frequency of HLA-DR1 and -DR4 in patients with both MS and bipolar disorder with a family history of affective disorders [5]. More recently, Bozikas et al. investigated this possible association based on the study of the HLA system in family members of a patient with both MS and bipolar disorder and family history of bipolar disorder. This study showed that HLA-DR2 haplotype appears to be a susceptible locus for bipolar disorder. These studies suggest that genes near the HLA region on chromosome 6 could be involved in the multifactorial pathogenesis underlying the clinical comorbidity of the two disorders [7]. Our patient in the third case has a family history of bipolar disorder and could therefore have a genetic susceptibility for this disorder. The hypothesis of a common vulnerability between MS and bipolar disorder could be advanced.

The manic symptoms may also be related to white matter lesions location [22]. Indeed, the orbitofrontal cortex is the main structure involved in regulating social behavior. Its disconnection from subcortical structures due to white matter damage in MS may explain, at least in part, the symptoms in the manic syndrome (exalted mood and disinhibition). This was noted in the third case as a new active lesion was found in the orbitofrontal cortex concomitant with the manic episode.

Bipolar disorder in MS patients is usually treated in the same way as in the general population. A treatment with mood stabilizers (sodium valproate, carbamazepine, and lithium) associated with atypical antipsychotics is generally effective on manic fits. This was the case in our three patients. The use of lithium must be with caution in patients with sphincter disorders since they tend to reduce their fluid intake and may thus have high serum levels of lithium approaching toxic doses [23]. Remission of psychiatric symptoms was noted using high doses of methylprednisolone even when fits were purely psychiatric [10]. This positive effect of corticosteroids against psychological fits supports the hypothesis of an organic cause for MS and bipolar comorbidity. It should be emphasized that the risk of exacerbation of psychiatric disorders using corticosteroids, which are not constant and occur more frequently in case of a discontinuous treatment, should not delay their use. In fact, patients manifesting psychiatric symptoms could still be treated with mood stabilizers, neuroleptics, or antidepressants with simultaneous steroid taper, as seen in Case  2. Thus, the main lesson for clinicians is that they should be aware of the possibility of steroid psychosis and be ready to treat it.

Interferon beta (IFN-*β*) treatment has been proposed to our patients to prevent relapses with good tolerance. Although initial studies have reported cases of suicide and depression in patients treated with IFN-*β*, none of the randomized controlled trials using standardized and validated measures of depression showed a significantly increased risk of depression in patients treated with IFN-*β* [24]. Other studies did not show a worsening of mood disorders in MS patients treated with IFN-*β* for a long period [25]. We can therefore conclude that the presence of major depression is not an absolute contraindication to treatment with IFN-*β*. Neurologists should, however, always be alert to the possible development of depression in all patients with MS, whether they are on disease-modifying treatment or not.

## 4. Conclusions

These three case reports highlighted the possible association between MS and bipolar disorder, an association that is still not well studied. We conclude that interferon beta treatments are well tolerated while high dose corticosteroids may induce manic fits. MS and bipolar association may be due to local MS-related brain damage or due to common genetic vulnerability. More studies focusing on specific response to treatment and genetic susceptibility are mandatory.
